# Potential Benefits of Sequential Inhibitor-Mutagen Treatments of RNA Virus Infections

**DOI:** 10.1371/journal.ppat.1000658

**Published:** 2009-11-13

**Authors:** Celia Perales, Rubén Agudo, Hector Tejero, Susanna C. Manrubia, Esteban Domingo

**Affiliations:** 1 Departamento de Virología y Microbiología, Centro de Biología Molecular “Severo Ochoa” (CSIC-UAM), Consejo Superior de Investigaciones Científicas (CSIC), Madrid, Spain; 2 Centro de Investigación Biomédica en Red de Enfermedades Hepáticas y Digestivas (CIBERehd), Barcelona, Spain; 3 Departamento de Bioquímica y Biología Molecular I, Universidad Complutense de Madrid, Madrid, Spain; 4 Centro de Astrobiología (CSIC-INTA), Madrid, Spain; Stanford University School of Medicine, United States of America

## Abstract

Lethal mutagenesis is an antiviral strategy consisting of virus extinction associated with enhanced mutagenesis. The use of non-mutagenic antiviral inhibitors has faced the problem of selection of inhibitor-resistant virus mutants. Quasispecies dynamics predicts, and clinical results have confirmed, that combination therapy has an advantage over monotherapy to delay or prevent selection of inhibitor-escape mutants. Using ribavirin-mediated mutagenesis of foot-and-mouth disease virus (FMDV), here we show that, contrary to expectations, sequential administration of the antiviral inhibitor guanidine (GU) first, followed by ribavirin, is more effective than combination therapy with the two drugs, or than either drug used individually. Coelectroporation experiments suggest that limited inhibition of replication of interfering mutants by GU may contribute to the benefits of the sequential treatment. In lethal mutagenesis, a sequential inhibitor-mutagen treatment can be more effective than the corresponding combination treatment to drive a virus towards extinction. Such an advantage is also supported by a theoretical model for the evolution of a viral population under the action of increased mutagenesis in the presence of an inhibitor of viral replication. The model suggests that benefits of the sequential treatment are due to the involvement of a mutagenic agent, and to competition for susceptible cells exerted by the mutant spectrum. The results may impact lethal mutagenesis-based protocols, as well as current antiviral therapies involving ribavirin.

## Author Summary

RNA viruses are associated with many important human and animal diseases such as AIDS, influenza, hemorrhagic fevers and several forms of hepatitis. RNA viruses mutate at very high rates and, therefore, can adapt easily to environmental changes. Viral mutants resistant to antiviral inhibitors are readily selected, resulting in treatment failure. The simultaneous administration of three or more inhibitors is a means to prevent or delay selection of resistant mutants. A new antiviral strategy termed lethal mutagenesis is presently under investigation. It consists of the administration of mutagenic agents to elevate the mutation rate of the virus above the maximum level compatible with virus infectivity, without mutagenizing the host cells. Since low amounts of virus are extinguished more easily, the combination of a mutagen and inhibitor was more efficient than a mutagen alone in driving virus to extinction. Here we show that foot-and-mouth disease virus replicating in cell culture can be extinguished more easily when the inhibitor guanidine is administered first, followed by the mutagenic agent ribavirin, than when both drugs are administered simultaneously. Interfering mutants that contribute to extinction were active in the presence of ribavirin but not in the presence of guanidine. This observation provides a mechanism for the advantage of the sequential versus the combination treatment. This unexpected effectiveness of a sequential treatment is supported by a theoretical model of virus evolution in the presence of the inhibitor and the mutagen. The results can have an application for future lethal mutagenesis protocols and for current antiviral treatments that involve the antiviral agent ribavirin when it acts as a mutagen.

## Introduction

The capacity of rapidly multiplying parasites to adapt to the changing environment of their host organisms is a formidable obstacle for the control of the diseases they bring about, and an unsolved problem in medical and veterinary practice. RNA viruses are remarkably adaptable due to their elevated mutation rates and quasispecies dynamics [1]–[4]. A means to compromise the replication of highly variable RNA viruses is to enhance their mutation rates above the maximum level compatible with expression of their genetic program and completion of their life cycle. The existence of an error threshold for genetic stability was predicted by quasispecies theory, that established a correlation between the average error rate and the complexity of the genetic information that can be reproducibly maintained [1]–[3],[5]. This concept has been supported by additional theoretical treatments [5]–[18]. RNA virus genomes and other RNA genetic elements have limited length (encoded information), and, accordingly, they tolerate the high mutation rates that they display during replication [19]–[21]. If in the course of virus replication the error rate is elevated above the error threshold, the result should be the extinction of the virus, in a process that has been termed lethal mutagenesis. Extensive experimental evidence has documented viral extinction upon replication of RNA viruses in the presence of mutagenic agents, in particular mutagenic nucleoside analogues [22]–[35].

In an exploration of variables that could influence extinction of the picornavirus foot-and-mouth disease virus (FMDV), it was noted that low viral fitness and low viral load favored extinction [33],[36]. As a consequence, combinations of mutagenic agents and antiviral inhibitors were more effective than mutagenic agents alone in driving viral populations towards extinction [36]–[39]. The advantage of a combination of a mutagenic agent and an antiviral inhibitor in lethal mutagenesis was in agreement with the requirement of combination treatments to delay or prevent selection of escape mutants when employing non-mutagenic antiviral inhibitors, as supported by theoretical studies and clinical observations [40]–[46].

The nucleoside analogue ribavirin [1-(β-D-ribofuranosyl)-1*H*-1,2,4-triazole-3-carboxamide] (R) is a licensed antiviral agent shown to be mutagenic for a number of RNA viruses [47]–[53], currently used in investigations on lethal mutagenesis. Viral mutants with decreased sensitivity to R have been described for several viruses, including the picornaviruses poliovirus (PV) and FMDV [54]–[59]. The presence of a mutation that decreased the sensitivity of FMDV to R resulted in extinction failure when the virus was replicated in the presence of high concentrations of R [60]. Extinction was achieved with an alternative mutagenic treatment with 5-fluorouracil [60]. Therefore, it is important to understand the molecular events that underlie virus extinction by mutagenic agents and to explore protocols that avoid selection of escape mutants.

Populations of FMDV and the arenavirus lymphocytic choriomeningitis virus (LCMV) subjected to lethal mutagenesis underwent a decrease of specific infectivity (with loss of infectivity preceding loss of RNA replication capacity), and pre-extinction, mutagenized RNA interfered with replication of infectious RNA [27],[61]. These results were further supported by simulations *in silico*, and suggested that interference by defective (non-infectious but replication-competent) genomes could contribute to loss of infectivity. These defective genomes were termed defectors, and their involvement in loss of infectivity led to the proposal of the lethal defection model of virus extinction, [27],[61], a model which was further strengthened by additional theoretical studies [62]. In agreement with this proposal, specific capsid and polymerase mutants of FMDV exerted complementation (at early times post-electroporation) and interference (at late times post-electroporation) when co-electroporated into cells together with infectious FMDV RNA [63]. Related FMDV mutants that were not competent in RNA replication did not exert any detectable interference [63]. In line with these observations, replication of drug-resistant poliovirus was inhibited by trans-acting, dominant negative mutants [64]. Therefore, three critical interconnected parameters have been identified as playing a role in viral extinction by lethal mutagenesis: mutation rate, interference by defector genomes, and viral load; the latter can be decreased by the presence of antiviral inhibitors [33],[61],[63],[65].

The combined use of mutagenic agents and antiviral inhibitors poses an evolutionary and a virological riddle to the system. First, the mutagenic agent, that is included in the drug cocktail, can favor the generation of viral mutants resistant to the inhibitors co-administered with the mutagen [36],[38]. Second, if intracellular replication is necessary for mutants to exert interference [63], the presence of an antiviral inhibitor together with the mutagenic agent may jeopardize the interfering activity of mutants by preventing or diminishing their accumulation during mutagenesis. However, if inhibitor-resistant mutants are generated, they could contribute defector genomes induced by the mutagen. Here we address this key issue using FMDV, specific interfering capsid and polymerase FMDV mutants, the inhibitor of FMDV replication guanidinium hydrochloride (GU), and R.

R eliminates FMDV from persistently and cytolytically infected cells in culture, and its mechanism of action includes mutagenesis of the viral RNA [59],[66],[67]. Failure to extinguish FMDV by a sequential treatment with either fluorouracil or R first, followed by GU, was systematically associated with selection of GU-resistant FMDV mutants, with amino acid replacements in non-structural protein 2C [36],[37],[60] (Perales et al., unpublished results). Here we show that, contrary to expectations from antiviral designs with non-mutagenic inhibitors, a sequential treatment first with GU and then with R can be more effective in driving the virus to extinction than the administration of GU and R in combination. Quantification of the interference exerted by specific FMDV mutants on the replication of standard FMDV suggests that the molecular basis of the advantage of the sequential treatment is that interfering FMDV mutants can exert a suppressive activity in the presence of R, when GU is not present. However, the presence of GU at the same time than R in the combination treatment would jeopardize the interfering activity of the mutants by inhibiting their replication, thereby contributing to virus survival. The results are supported by a theoretical model that predicts the evolution of a viral population in the presence of a mutagenic agent and an antiviral inhibitor. From a practical point of view, the proposed treatment protocol has the advantage that the simultaneous administration of two drugs is avoided, and that low mutagen doses may be sufficient to effect viral clearance.

## Results

### Effect of guanidine concentration on foot-and-mouth disease virus replication and selection of escape mutants

**Figure 1:**
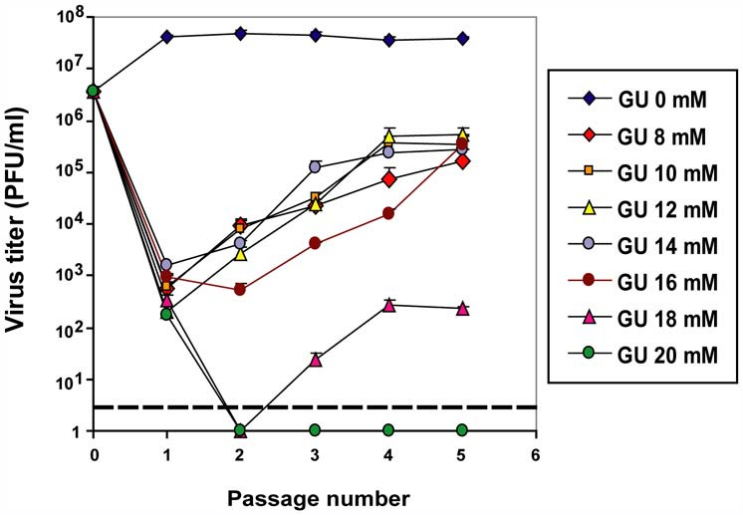
Effect of guanidine (GU) concentration on the yield of progeny FMDV pMT28. Passages were carried out by infecting 2×10^6^ BHK-21cells with virus pMT28, in the presence of increasing concentrations of GU, as indicated in the boxes on the right. The initial multiplicity of infection (MOI) was 1–2 PFU/cell. For subsequent passages, 0.2 ml of supernatant from the previous passage were used, resulting in MOIs of 10^−5^ to 10^−1^ PFU/cell in the presence of GU. Conditions for the infections, treatment with guanidine hydrochloride, and for determination of FMDV infectivity by plaque assay are detailed in Materials and Methods. The discontinuous line indicates the limit of measurement of infectivity values.

To compare a sequential versus combination treatment involving GU and R, we first determined the maximum GU concentration that permitted at least two passages of FMDV pMT28 (virus rescued from an infectious transcript; see Materials and Methods) with a decrease of viral load, prior to dominance of GU-resistant mutants [36],[37]. The results (Figure 1) indicate that there is a recovery of virus infectivity in the presence of GU concentrations ranging from 8 to 18 mM, but not 20 mM, and this is compatible with the dominance of GU-resistant mutants, as we have previously shown [36],[37]. GU concentrations of 16, 18 and 20 mM led to a decrease in virus progeny production for at least two consecutive passages, and, therefore, these GU concentrations were chosen to compare the efficacy of a sequential versus combination treatment involving GU and R.

### Benefits of inhibitor-mutagen sequential treatment versus inhibitor+mutagen combination treatment

**Figure 2:**
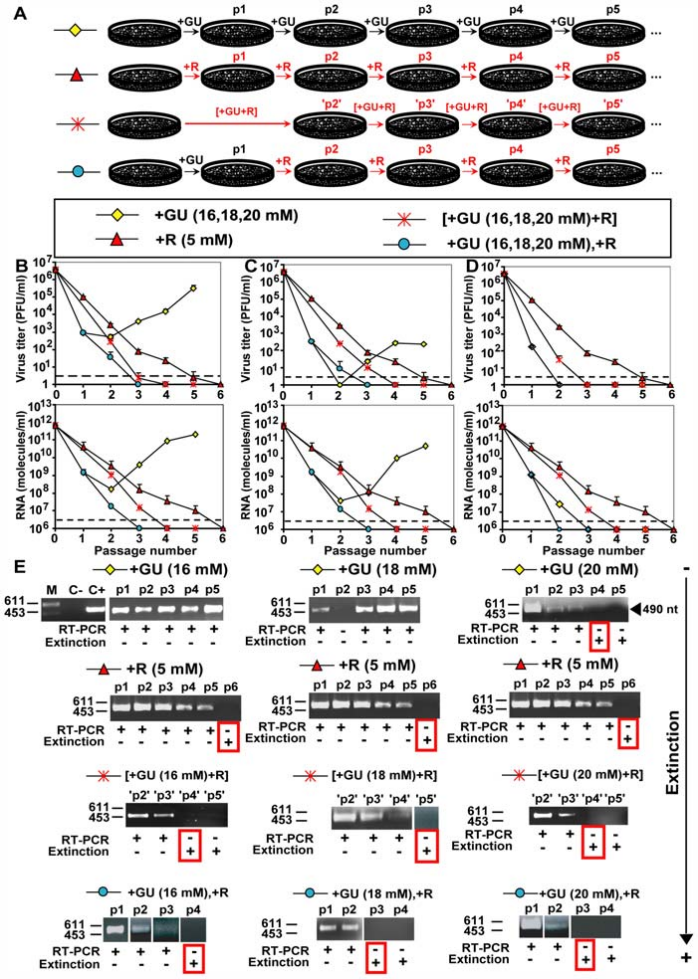
Sequential versus combination treatment with guanidine (GU), and ribavirin (R) on progeny production of FMDV pMT28. (A) Scheme of the passage protocol used for the different treatments compared in the present study. Passages that involve ribavirin (R) treatment are highlighted in red. Note that for comparison, one passage in the presence of the combination [+GU+R] was considered equivalent to the sequential +GU, +R passages; this is indicated with ‘p2’, ‘p3’, ‘p4’ in the series involving the combination treatment. (B, C, D) BHK-21 cells were infected with pMT28 in the presence of either GU [16, 18 or 20 mM], or R (5 mM), or the combination of GU and R. The only difference in the experiments described in (B), (C) and (D) is the concentration of GU, which was 16 mM for the assays in panels B, 18 mM in panel C and 20 mM in panel D. The combination treatment is indicated in brackets, while in the sequential treatment the order of administration of GU and R is separated by a comma. Passages were carried out by infecting 2×10^6^ BHK-21 cells with virus (0.2 ml of supernatant from the previous passage resulting in MOIs of 10^−6^ to 10^−1^ PFU cell), and infectivities and FMDV RNA levels were determined as detailed in Materials and Methods. The discontinuous lines in the virus titer and RNA quantification indicate the limit of measurement of infectivity values, and the limit of detection of FMDV RNA for real time PCR quantification, respectively. Each value represents the mean ± standard deviation (error bars) from triplicate determinations. (E) RT-PCR amplifications used to detect viral RNA in the cell culture supernatants, to monitor virus extinction. The sample analyzed and passage number (nomenclature of part A and B) are indicated above each panel. The presence or absence of a visible band after RT-PCR amplification, and the occurrence or not of extinction are indicated as + or −; the first passage in which extinction was observed is boxed in red. Molecular size markers (Hind III-digested Ø29 DNA; the corresponding sizes [base pairs] are indicated on the left); C-, negative control; amplification without RNA; C+, PCR positive control, are shown for the first panel only, but they were included in all the amplification assays. Conditions of the infections, mutagenic treatment, determination of infectivity, quantification of FMDV RNA, and RT-PCR amplification are detailed in Materials and Methods.

A sequential treatment first with GU and then with R was compared with treatment with R or GU alone, and with a combination treatment in which the inhibitor and the mutagenic agent were administered simultaneously. Drug doses and times of exposure were comparable in the different treatment protocols. To this aim, FMDV pMT28 populations were subjected either to one passage in the presence of GU (16, 18 or 20 mM) followed by three passages in the presence of R (5 mM), or to four passages in the presence of a combination of GU (16, 18 or 20 mM) and R (5 mM), or at least five passages in the presence of either R (5 mM) or GU (16, 18 or 20 mM) alone (Figure 2A). The sequential treatment is abbreviated as +GU,+R, and the combination treatment as [+GU+R]. The results (Figure 2B, C, D) show that the decrease in virus titer and in viral RNA levels was more rapid when GU was applied first and then R sequentially, as compared with the other treatment protocols. The second most effective treatment was achieved with the administration of GU and R at the same time, in a combination treatment [+GU+R]. At each passage, the virus titer and virus RNA levels attained one or more logarithms lower level with the sequential +GU,+R treatment than with the combination treatment (Figure 2B, C, D) (p<0.008 for virus titer in all cases; p<0.0001 for viral RNA levels in all cases; Student's t test). The decrease in virus titer and viral RNA levels was more accentuated as the GU concentration was increased. To ascertain that the decrease in the viral replication correlates with the extinction of the viral population, RT-PCR amplifications for every passage were performed. FMDV is considered extinct when no infectivity and no viral RNA can be detected in the cell culture supernatant, using a highy sensitive RT-PCR protocol, and after 3 blind passages in the absence of any drug (see Materials and Methods for further details). The advantage of the sequential treatment was evidenced by loss of FMDV-specific genetic material, indicative of virus extinction, at the same or at earlier passages for sequential than either combination treatment or treatment with GU or R alone (Figure 2E). The results using GU or R alone showed a recovery in virus titer and RNA levels for GU concentrations of 16 mM and 18 mM, whereas virus was not extinguished until passage 6 using R alone.

### Interference by specific FMDV mutants is exerted in the presence of ribavirin, but suppressed by GU

**Figure 3:**
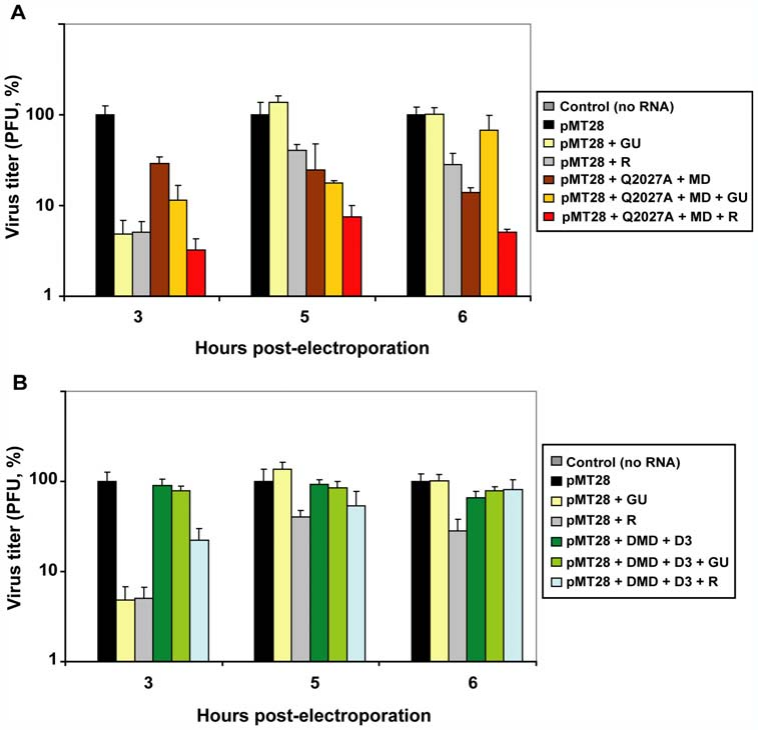
Effects of ribavirin (R) and guanidinium (GU) on the interference on FMDV replication exerted by capsid and 3D polymerase mutants. (A) Cells were mock-electroporated (control), or electroporated with 2 µg of pMT28 transcript (expresing FMDV C-S8c1), or co-electroporated with a mixture of transcripts obtained from pMT28 and from capsid mutant Q2027A and 3D polymerase mutant MD [63]. +R and +GU indicate the presence of R (1 mM) or GU (2 mM), respectively, as indicated in the box on the right. The ratio of pMT28 RNA to the total amount of mutant RNA was 1∶10, and the total amount of FMDV RNA was the same in each electroporation assay. Infectivity values were determined in triplicate at the indicated hours post-electroporation. Virus titers are expressed as percentage of the titers produced, relative to those produced by pMT28 RNA at the corresponding hours post-electroporation, taken as 100%. (B) Same as in (A) but using in the coelectroporations RNA from non-interfering polymerase mutants DMD and D3 [63]. In (A) and (B), no infectivity was obtained from the mock-electroporated samples (control box in dark grey, not visible). The origin of FMDV mutants, and procedures for titration of virus are detailed in Materials and Methods.

The advantage of the sequential +GU,+R over the combination [+GU+R] treatment was surprising in view of the previously established (and broadly accepted) requirement of a combination therapy to minimize or prevent selection of viral mutants resistant to antiviral agents [40]–[46]. We suspected that the critical difference could be the involvement of a mutagenic agent in the treatment. Mutants generated in the course of R mutagenesis may play an important role in the extinction of RNA viruses during lethal mutagenesis treatments [27],[62],[63]. Thus, one interpretation of the lower infectivity and viral loads as a result of the sequential +GU,+R treatment over the combination [+GU+R] treatment is that the interfering mutants generated by R-mutagenesis might have been suppressed by GU when this inhibitor is added at the same time. To investigate this possibility, we used previously characterized interfering and non-interfering capsid and polymerase mutants of FMDV [63]. BHK-21 cells were co-electroporated with standard, infectious FMDV RNA and combinations of either interfering FMDV mutants or of non-interfering FMDV mutants, in the absence or presence of GU and R. The concentrations of GU and R that decreased at least one logarithm the infectious progeny production at 3 hours post-electroporation were previously determined (data not shown). The results (Figure 3 and Table 1) show that the interfering combination of capsid mutant Q2027A and polymerase mutant MD exerted an interference in the presence of R at all times post-electroporation tested. In contrast, in the presence of GU, the interference decreased significatively at late times post-electroporation (Figure 3A). The average interference (of the values at 3, 5 and 6 h post-electroporation) exerted by the mutant combination Q2027A+MD was 81% in the absence of GU and 61% in the presence of GU. This decrease is statistically significant (p = 0.004 for the 20% decrease with a 95% confidence interval of 29.6% to 10.4%; Student's t test). In contrast, the average interference exerted by the same mutant combination was 81% in the absence of R and 94% in the presence of R. This increase is statistically significant (p = 0.005 for the 12% increase with a 95% confidence interval of 6.37% to 19.58%). When expressed as the interference measured at each time point (Table 1) the results indicate strong suppresion of interference by GU (but not by R) at the late time point at which the Q2027A+MD combination exerts its highest effect [63]. The results suggest that GU may inhibit replication of defector genomes, while R may contribute to the generation of additional defector genomes (see Discussion). No significant differences were observed in the virus titers produced in the presence of the combination of non-interfering mutants DMD+D3 in the presence and absence of GU (p = 0,5; Student's t test), as expected. The 2C-coding region of the progeny of the RNAs that were electroporated in the presence of GU was sequenced. No mutations associated with GU-resistance were detected in any sample. Thus, the decrease in interference in the presence of GU, upon replication immediately following electroporation, was not due to replication of GU-resistant mutants. These experimental results suggest that the mechanism by which the sequential treatment is more efficient than the combination treatment in decreasing the viral load and driving a virus towards extinction, is that in the sequential treatment the inhibitor cannot prevent replication of defector genomes that are generated by the mutagenic agent.

**Table 1:** Interference by specific FMDV mutant combinations.

Components present during FMDV (pMT28) genome replication^a^					Interference (%)^b^		
pMT28	Q2027+MD	DMD+D3	+GU	+R	3 h	5 h	6 h
+	−	−	−	−	NA^c^	NA^c^	NA^c^
+	+	−	−	−	72.0	75.0	86.0
+	+	−	+	−	88.5	82.2	32.8
+	+	−	−	+	96.7	92.5	94.9
+	−	+	−	−	ND^c^	ND^c^	34.2
+	−	+	+	−	21.7	14.2	21.6
+	−	+	−	+	77.8	32.5	13.6

a Interference was determined by measuring infectious progeny production after co-electroporation of pMT28 RNA alone (first row) or with mixtures of mutant FMDV RNAs (either Q2027A+MD or DMD+D3) in the absence (−) or presence (+) of guanidinium (GU) or ribavirin (R), as indicated. The origin of FMDV mutant RNAs and experimental procedures are detailed in Materials and Methods.

b Interference (%) is defined as [titer pMT28–titer pMT28 (plus indicated components)/titer pMT28]×100. Values are the average of three determinations, at 3 h, 5 h and 6 h post-electroporation. Virus titers (expressed as percentage of the titer obtained for pMT28 alone) and standard deviations are given in Figure 3.

c NA, not applicable; ND, not detectable.

### The advantage of sequential treatment is supported by a theoretical model

The lethal defection model of virus extinction was proposed on the basis of experimental results with FMDV and LCMV and of a computational model with virtual viable and defective genomes that replicated in the course of a persistent LCMV infection of BHK-21 cells [27]. Current evidence suggests that trans-active viral gene products harboring amino acid substitutions may impair functions of standard genomes whenever protein complexes are required for function (homo and hetero-polymers among viral proteins or between viral and host proteins). For further discussion of possible interference mechanisms, see [4],[25],[27],[61],[64],[65]. Using realistic parameters for viral genome replication and mutation rates, the model predicted a decisive participation of defector genomes in the extinction of the viable class of genomes [27],[60]. Therefore, we have now developed another model for the evolution of viral populations that replicate in serial cytolytic infections under increased mutagenesis, and in the presence of an inhibitor of viral replication. We have asked whether this new model predicts the experimental results reported here, in particular an advantage of sequential over combination treatment.

**Figure 4:**
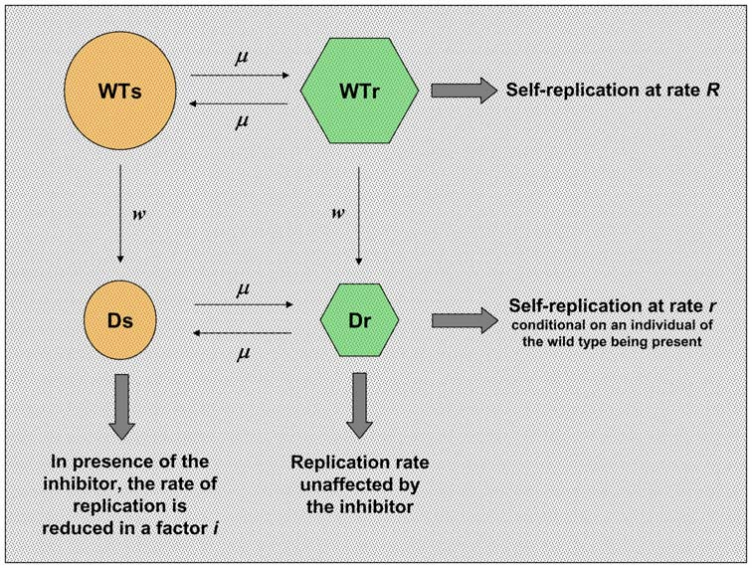
Schematic representation of the theoretical model for virus evolution. Four different viral types (circles and hexagons) describe the viral population: wild-type susceptible to the inhibitor (WTs), wild-type resistant (WTr), defective susceptible (Ds), and defective resistant (Dr). WTr are generated through mutation by the WTs type. Any wild-type individual can produce defectors under replication. The rates of generation of resistants and defectors are linked, such that increases in the mutation rate increase proportionally both rates (the former being substantially smaller than the latter). From the viewpoint of the virus, increased mutagenesis has two opposed effects: it promotes the appearance of resistant forms but at the same time enhances the appearance of defectors, which can induce extinction. Survival thus appears as a subtle balance between both trends.

The model considers four different types of individuals in the population: wild-type susceptible to the inhibitor (WTs), defective susceptible to the inhibitor (Ds), wild-type resistant to the inhibitor (WTr), and defective resistant to the inhibitor (Dr), but does not consider any direct interference exerted by the defective genomes on wild type replication (Figure 4). Individuals of the wild-type are able to replicate by themselves when they infect a cell; defective individuals cannot replicate in absence of wild-type individuals. WTs and Ds replicate more slowly in the presence than in the absence of the inhibitor, while WTr and Dr are not affected by the inhibitor. The natural mutation rate during genome replication is *w*, which for simplicity is equated to the rate of production of defective forms, generated upon replication of the wild type. Individuals resistant to the inhibitor are generated at a rate *μ = k w*, with *k = *10^−3^ (values for the coefficient *k* ranging from 10^−2^ to 10^−5^ do not affect the results qualitatively). Hence, an increase in the mutation rate has two effects: a larger number of defective genomes are generated, and the probability to develop resistance to the inhibitor increases (Figure 4).

We consider a total of *N* = 10^7^ cells which can be infected at each passage. The maximum number of virions entering a cell is one. The initial virus replicates inside the cell for a number of cycles *G* = 5. When the inhibitor is absent, the number of progeny genomes per parental genome is given the value *R* = 1.5 for wild-type individuals, and *r* = 1 for defective individuals. When the inhibitor is present, the replicative parameters *R* and *r* are multiplied by a factor 0<*i*<1. The natural mutation rate is set to *w* = 0.05 in the examples that follow. At passage 0, the population is composed only of individuals sensitive to the inhibitor; resistant individuals will be generated by mutation. Replication inside each cell follows a deterministic process according to the above description. Let us call WTs(*g*), Ds(*g*), WTr(*g*), and Dr(*g*) the four populations at replication cycle *g*. In one replication cycle, the number of particles produced is

WTs(*g*+1) = *R i* (1−*μ−w*) WTs(*g*)

WTr(*g*+1) = *R* (1−*w*) WTr(*g*)+*R i μ* WTs(*g*)

Ds(*g*+1) = *i* Ds(*g*)+*R i w* WTs(*g*)

Dr(*g*+1) = Dr(*g*)+*R w* WTr(*g*)

We define the factor of inhibition (to be given in percent) as *f_i_* = 100 * (1−*i*), such that *f_i_* = 0% indicates no inhibition (in the equations above, parameter *i* = 1) and complete inhibition (or forbidding replication), corresponds to *f_i_* = 100% (*i* = 0). The total population produced by the cell in *G* viral replication cycles is the result of iterating *G* times the equations that define the model. The total viral population *P* after one passage is the sum of the production by the *N* cells. This final viral population is then used to seed a new ensemble of *N* cells, to start the next passage. The average number of virions entering each cell is *P/N*, unless it exceeds one (which is the maximum we allow in these examples).

**Figure 5:**
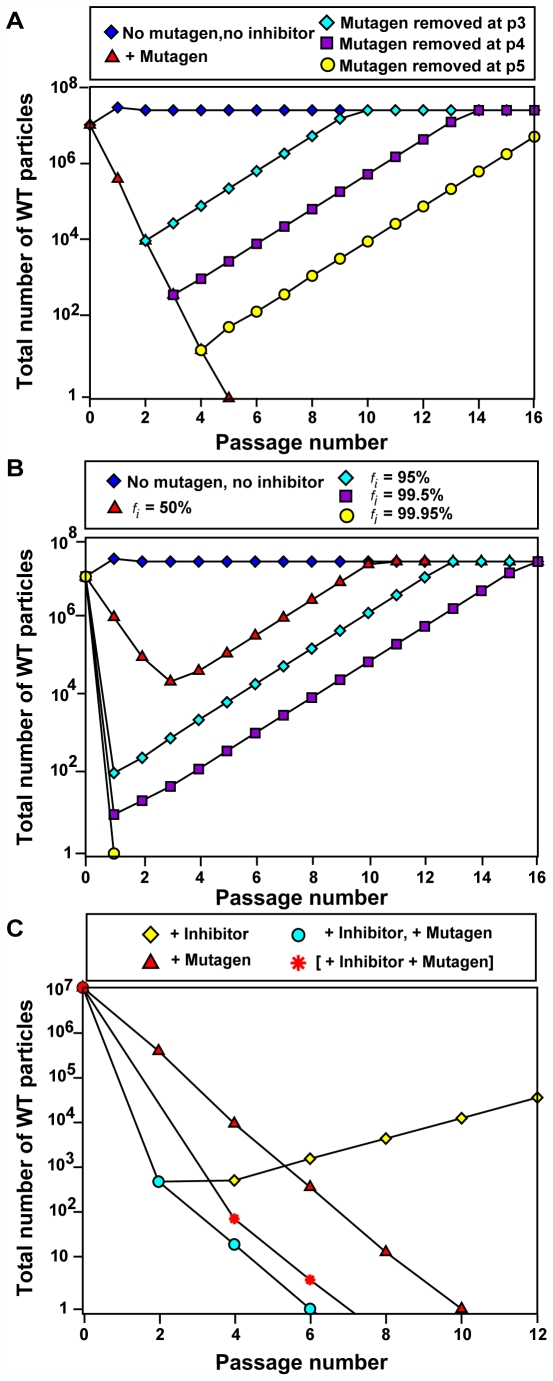
Dynamics of the model system in situations analogous to the experimental setting. Total number of wild-type individuals (either susceptible or resistant) as a function of the passage number. Parameters are *N* = 10^7^, μ = 10^−3^, *w* = 0.005 (in the absence of mutagen), *w* = 0.6 (in the presence of mutagen), *G* = 5. (A) Behavior of the population in the presence of mutagen, showing the high yield attained under the natural mutation rate *w* = 0.05 and the recovery of the population when the mutagen acts during a limited number of passages. For *w* = 0.6, extinction supervenes at passage 5. (B) Effect of different amounts of inhibitor in the population. This figure is analogous to Figure 1. If the amount of inhibitor is not high enough to guarantee extinction, resistant forms appear and are subsequently fixed in the population, which reaches a level of viral yield comparable to that in the absence of inhibitor after a sufficiently large number of passages. (C) Effect of different therapies on the fate of the population. The situations represented are analogous to those in Figure 2 (B–D), as described in the legend. Note the different effectiveness of the alternative treatments to achieve an undetectable number of WT particles.

A natural population evolves through passages with a mutation rate *w* = 0.05, and in the absence of inhibitor (*i* = 1). The addition of a mutagen changes the mutation rate to a higher value of *w*, while the addition of an inhibitor diminishes the replication rate of susceptible particles in an amount *i<*1. The example given in Figure 5A demonstrates the dynamics of the model in some of the experimental situations reported in the manuscript. When no mutagen and no inhibitor are present, the population evolving under the natural parameters reaches a high titer (measured as the number of wild-type individuals). When a mutagen is added (in practice, the value of *w* is set to 0.6), extinction after several passages occurs. Different curves show how the population recovers when the treatment with the mutagen is interrupted beginning at passages two, three, and four. This behavior is that observed when GU-escape mutants are selected in populations treated with a mutagen and GU ([36],[37],[60]; Perales et al., unpublished results).

In the second case (Figure 5B), we describe the dynamics when different amounts of inhibitor are used, analogous to the experimental assays presented in Figure 1. Increasing concentrations of inhibitor result in a gradually steeper decrease in the number of progeny particles. However, except for the highest inhibitor concentration tested, resistant mutants are selected, and progeny production finally reaches the level attained in the absence of inhibitor. The simulation agrees with the experimental results (compare Figure 1 and Figure 5B).

Finally, in Figure 5C we represent the dynamics of the population under different combinations of inhibitor and mutagen. The examples are chosen to mimic the experiments presented in Figure 2. Sequential therapy causes extinction faster than combination therapy, and a contributing factor is the appearance and fixation of resistant mutants during the combination treatment, promoted by the mutagen. The superiority of sequential therapy is observed over a broad range of parameters, though it is not generic for this model. The role played by parameters such as the replication rate of the wild type, the number of replication cycles inside a cell, or the relative effect of mutagen and inhibitor and how they interact when applied jointly will be explored in future studies (see also Discussion). Both, the experimental results and the theoretical model (for the parameters used) suggest an approximately ten-fold increase in the viral yield in the case of combination therapy compared to sequential therapy.

## Discussion

One of the major consequences of quasispecies dynamics for pathogenic RNA viruses is that subpopulations of viruses from the mutant spectra, that harbor mutations that decrease the sensitivity to antiviral inhibitors, are rapidly selected (review in [68]). Lethal mutagenesis exploits high mutation rates of RNA viruses [19],[20] to increase the average error rate during viral replication even further, until meaningful genetic information and viral functions deteriorate, and the virus is extinguished [22]–[35].

New nucleoside analogues are currently investigated as possible virus-specific mutagenic agents that could be included in lethal mutagenesis protocols [22], [29], [35], [69]–[71]. In addition to safety issues concerning adverse activity on the host cells (related to mutagenesis of cellular DNA, or other effects), the administration of virus-specific mutagenic base or nucleoside analogues requires careful consideration of protocols when antiviral inhibitors are co-administered with the mutagenic agents [72]. This has become particularly relevant with recent observations that suggest that replication-competent subsets of defective viral genome subpopulations termed defectors may participate in the process of viral extinction [27], [61]–[63]. Interference by defectors was specific for their corresponding standard viruses, was not due to induction of IFN or other unspecific cellular effectors, and it required replication of the interfering genomes [27],[61],[63]. In consequence, the presence in the infected cell of a mutagenic nucleotide together with an antiviral inhibitor may jeopardize extinction because the inhibitor will reduce replication of interfering genomes. This has been addressed experimentally in the present study, and, indeed, GU, but not R, attenuated the interfering activity exerted by a combination of a polymerase and capsid mutant of FMDV. In agreement with the attenuation of interference by GU, the decrease in FMDV infectivity, viral load and attainment of viral extinction are more effective with a sequential +GU,+R treatment than with the combination treatment [+GU+R], or treatment with R or GU alone (Figures 1 and 2). The differences were investigated using a constant R concentration of 5 mM, and GU concentrations in the range of 16 mM to 20 mM. Although eventually all treatment regimes achieved extinction (Figure 2C), the sequential +GU,+R treatment led earlier to low infectivity and RNA levels than the corresponding combination treatment (Figure 2B). In an *in vivo* scenario of application of lethal mutagenesis, an earlier and sustained decrease in viral load may provide the host immune response with an opportunity to effect viral clearance. An added benefit of a sequential treatment is that toxicity or antagonistic effects, additional to suppresion of interference, derived from simultaneous administration of two drugs, are avoided.

It must be stressed that the benefits of a sequential treatment do not hold for standard non-mutagenic antiviral agents, for which combination treatments are essential to prevent selection of inhibitor-resistant mutants [40],[41],[73]. According to our experimental and theoretical results there are two key influences that favor the inhibitor-mutagen sequential treatment, and that do not operate when only non-mutagenic inhibitors are involved. One is that the mutagenic agent increases the probability of selection of inhibitor-escape mutants, and this probability increases with the viral load. The second influence is that the interfering activity of defector genomes is important to drive the population towards extinction. The administration of the inhibitor will produce a decrease in viral load, that will render the system more susceptible to mutagenesis-mediated extinction, allowing expression of interfering activities associated with the mutagenized spectrum of mutants [33]. No mutations in the 2C-coding regions that confer resistance to GU have been detected in passage 2 of sequential or combination treatment in the presence of 16 mM GU (experiment of Figure 2). Thus, GU-escape mutants were not a factor in the disadvantage of the combination treatment. At present, we cannot exclude that other mechanisms may also contribute to the observed benefits of a sequential inhibitor-mutagen treatment.

The experimental results are supported by a simple model of viral evolution taking into account the minimal ingredients that describe the experimental system. Our numerical results indicate that four different viral types, as discussed, are essential to reproduce the observed dynamics. According to the model, the fast generation and fixation of resistant mutants (controlled by parameter μ, and the number *G* of replication cycles inside the cell) is the essential mechanism conferring advantage to sequential therapy. In the absence of a mutagenic activity, the model predicts benefits of combination therapy, as in previous models of virus dynamics in connection with drug therapy [40]. Future work will explore the range of parameters that provide the strongest advantages to either therapy, as well as the relevance of other dynamical rules (such as the inclusion of lethal mutations or more detailed relationships between genomic mutations and their effect on fitness) in the behavior of the model system. For example, the current model predicts that as the interfering activity of defectors increases, the advantage of the sequential over the combination treatment is gradually lost (Manrubia et al. unpublished results). It is not known why under strong suppression (unrealistic for mutants generated by random mutagenesis within the quasispecies) the response of the system is that expected for administration of classical, non-mutagenic antiviral inhibitors, and this point is under investigation.

The results reported here can impact current antiviral therapies that involve R, such as the combination of pegylated IFN-α (PEG-IFN-α) and R for treatment of human HCV infections. In these treatments, IFN can have multiple effects at the level of the entire organisms, and also it is not clear whether ribavirin acts as a mutagenic agent, which would imply a lethal mutagenic action, or by other mechanisms, or combination of mechanisms [74]–[82]. For patients who respond to IFN-α treatment, and their HCV load is reduced, a sequential +IFN-α (or PEG- IFN-α),+R treatment may be advantageous over the corresponding combination treatment. Our experimental and theoretical results predict this to be true to the extent that R acts as a mutagenic agent for HCV, and that mutagenesis is its major mechanism of action against HCV *in vivo*. However, R has multiple affects on cell metabolism [83]–[87] it is not easy to assess which is the contribution of mutagenesis and defector genomes, in the course of treatments of HCV infections [34], [66], [87]–[90]. The first clinical trials, that consisted in the administration of recombinant IFN-α to chronic non-A, non-B hepatitis, resulted in improvement of aminotransferase levels and liver histology [91]. Subsequent treatments, once HCV had been identified, involved IFN-α alone or in combination with R [92],[93]. Some early clinical trials documented benefits of a combination [IFN-α,+R] treatment versus either treatment with IFN-α alone, or sequential treatment first with R and then with IFN-α, in chronic HCV infections [94]. More recent trials established a higher efficacy of PEG-IFN-α over conventional IFN-α, in combination treatments with R [95]. Other trials have compared IFN-α or R monotherapy with combination therapies or sequential therapies involving administration of R first [74], [96]–[99]. In a trial for the treatment of chronic HCV and HIV-1 in doubly-infected hemophiliacs, IFN-α-2b was administered as monotherapy for one month, and then oral R was added to the treatment [100]. To our knowledge, no systematic trials have involved sequential treatment with IFN-α first, and then with R alone, precisely the protocol predicted to be more effective, according to our results.

Exploration of sequential inhibitor-mutagenic treatments for HCV infections may become more relevant in the face of the new generation of specific inhibitors of HCV replication, now at different stages of development for clinical practice [101],[102]. Our prediction should hold also for treatment of other chronic viral infections, such as human hepatitis B virus for which both mutagenic nucleoside analogues and non-mutagenic inhibitors are available for treatment [103],[104]. It is obvious, however, that because of the complexities involved in pharmacological activities *in vivo* [83]–[87], experiments with animal models are needed to explore whether results *in vivo* will be those predicted by our model experiments in cell culture, and by the theoretical study.

## Materials and Methods

### Cells and viruses

The origin of BHK-21 cells and procedures for cell growth in Dulbecco's modification of Eagle's medium (DMEM), and for plaque assays in semisolid agar have been previously described [105],[106]. The viruses used in the experiment are the following: FMDV C-S8c1 is a plaque-purified derivative of serotype C isolate C1 Santa Pau-Sp70 [106]. An infectious clone of FMDV C-S8c1, termed pMT28 was constructed by recombining into a pGEM-1 plasmid subclones that represented the C-S8c1 genome, as described [107],[108]. Thus, FMDV pMT28 used in the experiments is the progeny of infectious transcripts that express the standard FMDV C-S8c1. Capsid (Q2027A) and polymerase (MD, DMD and D3) mutants were previously described, and characterized biologically and with an interference index [63],[109]. To control for the absence of contamination, mock-infected cells were cultured and their supernatants were titrated in parallel with the infected cultures; no signs of infectivity or cytopathology in the cultures or in the control plaque assays were observed in any of the experiments.

### Treatment with guanidine hydrochloride (GU)

A solution of guanidine (GU) in DMEM was prepared at a concentration of 50 mM, sterilized by filtration, and stored at 4°C. Prior to use, the stock solution was diluted in DMEM (Dulbecco's modification of Eagle's medium) to reach the desired concentration. For infections of BHK-21 cells with FMDV in the presence of GU, no pretreatment of the cell monolayer with GU was performed. After addition of FMDV and washing of the cell monolayers, infections were allowed to continue in the presence of GU. For each passage 2×10^6^ BHK-21 cells were infected with supernatant of virus from the previous passage (0.2 ml), and the infection allowed to proceed for about 24 h. The multiplicity of infection (MOI) ranged from 1×10^−5^ to 1×10^−1^ PFU/cell, and the MOI for each passage can be calculated from the infectivity values given in the experiment (Figure 1). Infections in the absence of GU, and mock-infected cells were maintained in parallel; no evidence of contamination of cells with virus was observed at any time.

### Treatment with ribavirin (R)

A solution of R in PBS was prepared at a concentration of 100 mM, sterilized by filtration, and stored at −70°C. Prior to use, the stock solution was diluted in DMEM to reach the desired R concentration. For infections in the presence of R, cell monolayers were pretreated during 7 h with 5 mM R prior to infection. FMDV C-S8c1 was passaged serially in the absence or in the presence of R (5 mM). After addition of FMDV and washing of the cell monolayers, the infection was allowed to proceed in the presence of the same concentration of R. For each passage 2×10^6^ BHK-21 cells were infected with supernatant of virus from the previous passage (0.2 ml) and the infection continued for about 24 h. The multiplicity of infection (MOI) ranged from 5×10^−6^ to 1×10^−1^ PFU/cell, and the MOI for each passage can be calculated from the infectivity values given for each experiment (Figures 2B, C, D). The passage experiments described in Figures 1 and 2 occurred over a 24-hour period, and therefore each passage is comprised of multiple replication cycles. Infections in the absence of R, and mock-infected cells were maintained in parallel; no evidence of contamination of cells with virus was observed at any time.

### Assessment of FMDV extinction

FMDV was considered extinct when no virus infectivity and no viral RNA that could be amplified by a highly sensitive RT-PCR protocol, could be demonstrated neither in the supernatant of the passage that harbors the putatively extinghuished virus, nor after 3 blind passages in BHK-21 cells, in the absence of any drug. Multiple highly sensitive RT-PCR amplification reactions that yield short cDNAs were carried out to ascertain extinction. Some of the gels that did not show a visible band were overexposed to ascertain absence of detectable DNA. These criteria to consider FMDV extinct [33],[36],[37],[59],[66] have now been extended to show that no infectivity or RT-PCR amplifiable material can be retrieved after passaging of the cells that harbor the putatively extinguished virus. This extension was prompted by the observation that FMDV subjected to hundreds of plaque-to-plaque transfers could lose capacity to form plaques and yet maintain intracellular RNA [110]. It should be noted that infectivity below the level of detection did not necessarily imply extinction (see Figure 2).

### RNA extraction, RNA quantification, cDNA synthesis and PCR amplification

Viral RNA was extracted from the medium of infected cells using Trizol (Invitrogen) as previously described [110]. Reverse transcription was performed with AMV reverse transcriptase (Promega), and PCR amplification was carried out using Expand High Fidelity (Roche), as specified by the manufacturers. The 3D-coding region was amplified using as primers oligonucleotide A2SacI (5′- CACACATCGACCCTGAACCGCACCACGA; sense orientation; the 5′ nucleotide corresponds to genomic residue 6581), and oligonucleotide AV4 (5′- TTCTCTTTTCTCCATGAGCTT; antisense orientation; the 5′ nucleotide corresponds to genomic residue 7071). Genomic residues are numbered as described in [111]. Amplification products were analyzed by agarose gel electrophoresis using *HindIII*-digested Ф-29 DNA as molar mass standards. Negative controls (amplifications in the absence of RNA) were included in parallel to ascertain absence of contamination by template nucleic acids.

### Quantification of FMDV RNA

Real time quantitative RT-PCR was carried out using the Light Cycler RNA Master SYBR Green I kit (Roche), according to the instructions of the manufacturer and as described previously for FMDV RNA [110]. The 2C-coding region was amplified using as primers oligonucleotide 2CR2 (5′- GGCAAACCCTTCAGCAGTAAG; sense orientation; the 5′ nucleotide corresponds to genomic residue 4924), and oligonucleotide 2CD3 (5′- CGCTCACGTCGATGTCAAAGTG; antisense orientation; the 5′ nucleotide corresponds to genomic residue 5047). Quantification was relative to a standard curve obtained with known amounts of FMDV RNA, synthesized by *in vitro* transcription of FMDV cDNA (plasmid pMT28). The specificity of the reaction was monitored by determining the denaturation curve of the amplified DNAs. Negative controls (without template RNA and RNA from mock-infected cells) were run in parallel with each amplification reaction, to ascertain absence of contamination with undesired templates.

### Transcription of viral RNA and electroporation of BHK-21 cells

Plasmid DNA was linearized by cleavage with the appropiate restriction enzymes (pO_1_K/C-S8c1, and the capsid mutant plasmids with *Hpa* I and pMT28 derivates with *Nde* I, as previously described [63]). Then, the plasmids were purified by Wizard PCR Preps DNA purification resin (Promega), and dissolved in RNase-free water. FMDV RNA was transcribed from the linearized plasmids by using the Riboprobe *in vitro* transcription system (Promega). The mixture contained transcription buffer (Promega), 10 mM dithiothreitol, 0.48 units/µl RNasin, 1 mM each of ribonucleoside triphosphates, 4 ng/µl linearized plasmid DNA, and 0.3 or 0.4 units/µl SP6 or T7 RNA polymerase; it was incubated for 2 h at 37°C. The RNA concentration was estimated by agarose gel electrophoresis, with known amounts of rRNA as markers.

To electroporate BHK-21 cells with RNA transcribed *in vitro*, subconfluent cells were harvested, washed with ice-cold phosphate-buffered saline (PBS), and resuspended in PBS at a density of about 2.5×10^6^ cells/ml. Aliquots (50–80 µl) of transcription mixture with the apropiate amount of RNA were added to 0.4 ml of cell suspension, and the mixtures were transferred to 2 mm electroporation cuvettes (Bio-Rad). Electroporation was performed at room temperature by two consecutive 1.5 kV, 25 µF pulses using a Gene Pulser apparatus (Bio-Rad), as described [63]. As control, BHK-21 cells were electroporated with 50–80 µl of transcription mixture in PBS to monitor absence of contamination. The cells were then resuspended in growth medium and seeded onto culture plates. At 3, 5 and 6 hours post-electroporation, samples of cells and culture medium were withdrawn and after three cycles of freezing at −70°C and thawing at room temperature, the lysate was stored at −70°C. Mock-coelectroporated cultures were treated in parallel and served as control in the titration of virus infectivity. No evidence of viral contamination was obtained in any of the experiments.
